# Synthesis and Anti-Human Immunodeficiency Virus Type 1 Activity of (*E*)-*N-*Phenylstyryl*-N-*alkylacetamide Derivatives

**DOI:** 10.3390/molecules14093176

**Published:** 2009-08-26

**Authors:** Pi Cheng, Ji-Jun Chen, Ning Huang, Rui-Rui Wang, Yong-Tang Zheng, Yi-Zeng Liang

**Affiliations:** 1 School of Chemistry and Chemical Engineering, Central South University, Changsha 410083, China; 2 State Key Laboratory of Phytochemistry and Plant Resources in West China, Kunming Institute of Botany, the Chinese Academy of Sciences, Kunming 650204, China; 3 Laboratory of Molecular Immunopharmacology, Key Laboratory of Animal Models and Human Diseases Mechanisms, Kunming Institute of Zoology, the Chinese Academy of Sciences, Kunming 650223, China

**Keywords:** (*E*)-*N-*phenylstyryl*-N-*alkylacetamides, synthesis, reverse transcriptase, anti-HIV-1 activity

## Abstract

A series of (*E*)-*N-*phenylstyryl*-N-*alkylacetamides, **5**, were synthesized by direct reduction-acetylation of β-arylnitroolefins, followed by *N*-alkylation. The title compounds were characterized by ^1^H-NMR, EIMS and IR analysis. All the synthesized compounds were assayed as HIV-1 non-nucleoside reverse transcriptase inhibitors. A SAR study revealed that when group R^1^ in **5** was *ortho*-substituted, the resulting compounds showed better inhibitory activities against HIV-1 RT. Among the tested compounds, **5i** (R^1^ = 2-Br, R^2^ = 3,5-difluorobenzyl) exhibited the highest enzyme activity, with a 88.89% inhibitory ratio against HIV-1 reverse transcriptase at the tested concentration. Further cell-based anti-HIV-1 assays showed that compound **5i** exhibited a SI value of 29 with an EC_50 _value of 4 μM in C8166 cells.

## 1. Introduction

Acquired immunodeficiency syndrome (AIDS), a disease resulting from infection with Human Immunodeficiency Virus type 1 (HIV-1), is one of the world’s most serious health problems. Three essential enzymes are encoded by the HIV-1 *pol* gene: (1) HIV-1 reverse transcriptase (RT), (2) integrase (IN), and (3) protease (PR). These three enzymes compose the most important and validated targets for developing novel antiretroviral agents [1,2,3,4]. Among RT inhibitors, non-nucleoside reverse transcriptase inhibitors (NNRTIs) remain a high priority for medical research because of their structural diversity [5,6,7,8,9].

**Scheme 1:**
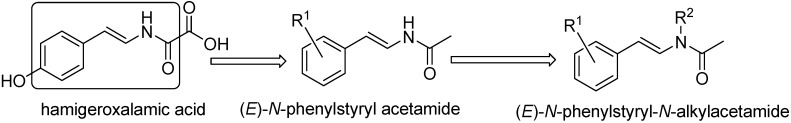
Structure of hamigeroxalamic acid, (*E*)-*N*-phenylstyryl acetamide and (*E*)-*N-*phenylstyryl*-N-*alkylacetamide.

Structure simplification of natural product has provided us an efficient way to find new and less toxic anti-HIV-1 lead compounds [10]. In a previous paper [11], we reported that (*E*)-*N*-phenylstyryl acetamides (Scheme 1), which contain the basic structural skeleton of hamigeroxalamic acid, could exhibit HIV-1 RT and showed for the first time moderate anti-HIV-1 activities in cell culture. Fundamental structure-activity relationships (SAR) were revealed and it was proposed that when group R^1^ was an *ortho*-substituent, the resulting compounds showed better inhibitory activities against HIV-1 RT. The *ortho*-substituted analogues thus represent a new template for further structure modification with a view to finding more active anti-HIV-1 analogues.

In this paper, a series of (*E*)-*N-*phenylstyryl*-N-*alkylacetamides (Scheme 1) were synthesized and evaluated *in vitro* as NNRTIs of HIV-1. Besides, syncytium reduction and cytotoxicity activities of the title compounds in cell based assay are provided. Detailed synthetic procedures and the anti-HIV-1 activities of these *N-*phenylstyryl*-N-*alkylacetamides are reported. 

## 2. Results and Discussion

### 2.1. Chemistry

**Scheme 2:**
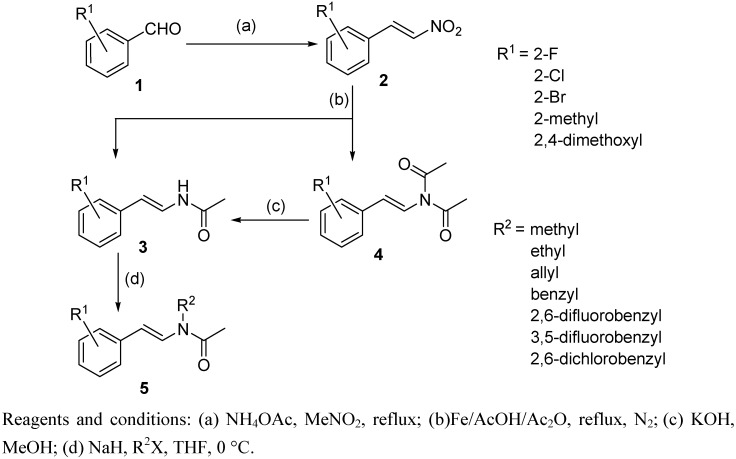
Synthetic route for (*E*)-*N-*phenylstyryl*-N-*alkylacetamides (**5**).

The synthetic route to the (*E*)-*N-*phenylstyryl*-N-*alkylacetamides **5** are shown in Scheme 2. First, (*E*)-β-nitroolefins **2** were produced by refluxing various benzaldehydes with ammonium acetate and nitromethane. Compounds **3** were synthesized through a one-pot reduction of compounds **2** using Fe/AcOH/Ac_2_O. In this reaction, diacetyl compounds **4** were isolated as byproducts. These could also be converted into compounds **3 **by treatment of potassium hydroxide. Treatment of compounds **3** with sodium hydride and various alkyl halides provided the title compounds **5** in moderate yields. Structures of groups R^1^ and R^2^ were given in Scheme 2, and R^1^ were mainly substituted in the *ortho*-position on the aromatic ring.

**Figure 2:**
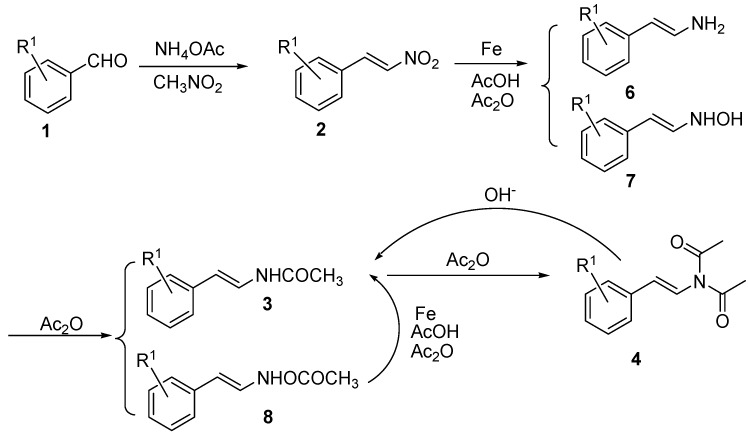
One-pot synthesis of *N*-phenylstyryl acetamides **3**.

A possible mechanism for the one-pot synthesis of (*E*)-*N*-phenylstyryl acetamides **3** by direct reduction-acetylation of (*E*)-β-phenyl-nitroolefins **2** is illustrated in Figure 2.

Compounds **2** would first be reduced to compounds **6** and/or **7**. Compounds **7 **would be trapped in the presence of acetic anhydride to form oxime acetates **8,** as would compounds **6**, to yield the desired (*E*)-*N*-phenylstyryl acetamides **3**. Oxime acetates **8** should be in turn reduced to **3** under the reaction conditions. Compounds **3 **might be further acetylated to the diacetyl compound **4**, a reaction that could be reversed by treatment with methanolic potassium hydroxide. It was worth noting that the *trans*-conformation of the carbon-carbon double bonds was maintained in this reduction–acetylation reaction.

**Table 1:** Structures and anti-HIV-1 activities of (*E*)-*N-*phenylstyryl*-N-*alkylacetamides **5**.^a^

Entry	R^1^	R^2^	RT inhibition (%)^b^	IC_50_^c^ (μM)	EC_50_^d^ (μM)	CC_50_^e^ (μM)	SI^f^ (CC_50_/EC_50_)
**5a**	3-Br	methyl	45.42	ND	128 ± 20	409 ± 106	3.2
**5b**	4-Br	methyl	16.59	ND	ND	ND	ND
**5c**	2-Br	methyl	85.27	339 ± 107	60 ±10	>789	>13.2
**5d**	2-Br	ethyl	46.37	ND	48 ± 8	358 ± 88	7.5
**5e**	2-Br	allyl	47.76	ND	75 ± 13	337 ± 154	4.5
**5f**	2-Br	acetyl	60.55	320 ± 22	8 ± 1	85 ± 17	10.6
**5g**	2-Br	benzyl	47.56	ND	9 ± 2	51 ± 13	5.7
**5h**	2-Br	2,6-difluoro-benzyl	57.33	295 ± 9	29 ± 4	172 ± 73	5.9
**5i**	2-Br	3,5-difluoro-benzyl	88.89	97 ± 13	4 ± 0.5	116 ± 13	29
**5j**	2-Br	2,6-dichloro-benzyl	30.34	ND	49 ± 10	190 ± 36	3.9
**5k**	2-Cl	methyl	75.22	434 ± 44	96 ± 20	441 ± 18	4.6
**5l**	2-Cl	ethyl	59.10	521 ± 11	91 ± 15	424 ± 22	4.7
**5m**	2-Cl	allyl	29.95	ND	118 ± 28	536 ± 41	4.5
**5n**	2-F	methyl	61.69	477 ± 107	130 ± 33	664 ± 79	5.5
**5o**	2-F	allyl	38.51	ND	261 ± 40	538 ± 138	2.0
**5p**	2-methyl	methyl	79.80	487 ± 27	116 ± 11	609 ± 177	5.2
**5q**	2-methyl	allyl	40.30	ND	273 ± 43	>930	>3.4
**5r**	2,4-dimethoxy	methyl	69.45	373 ± 40	32 ± 7	256 ± 35	8.0
**5s**	2,4-dimethoxy	ethyl	34.73	ND	19 ± 3	112 ± 10	5.9
**5t**	2,4-dimethoxy	allyl	36.62	ND	55 ± 4	290 ± 79	5.3
AZT^g^			ND^i^	ND	1.08×10^-^^2^	>509	>47130
PFA^h^			96.46	5	ND	ND	ND

^a ^All data represent mean values for at least two separate experiments. ^b ^All compounds were tested at 200 μg/mL. ^c^ concentration that inhibit 50% HIV-1 RT. ^e^EC_50_ = Effective concentration required to protect C8166 cells against the cytopathicity of HIV-1_Ⅲ__B_ by 50%. ^e^CC_50 _= Cytostatic concentration required to reduce C8166 cell proliferation by 50%. ^f^ Selectivity index: CC_50_/EC_50_ ratio. ^g^AZT was used as positive control. ^h^PFA: Foscarnet was tested at 20 μg/mL as positive control. ^i^ ND: not determined.

### 2.2. Biological activity

(*E*)-*N-*phenylstyryl*-N-*alkylacetamides **5** were assayed for their inhibitory ratio against HIV-1 RT at 200 μg/mL. As outlined in Table 1, **5a** and **5b **showed significantly less enzymatic activity, compared with **5c**, which suggests that the presence of an *ortho-*substituent on the aromatic ring in the *N*-methyl derivatives appears to increase enzyme inhibition. This positive influence of a group in *ortho* position as to compounds **3 **was already reported in our previous paper [11].

In some new developed NNRTIs such as the oxindole [12] and benzimidazole-2-one [13] derivatives, NH unit in the amide is a proton-donating group in the hydrogen bond formation process between the inhibitor and HIV-1 RT based on detailed docking studies, and *N*-alkyl analogues would suffer dramatic loss of activities. In our experiment, the SAR of compounds **5 **against HIV-1 RT was similar to that of compounds **3** — the *ortho* substituted compounds possessed higher enzymatic activity. Furthermore, compounds **5** possessed higher inhibitory ratio against HIV-1 RT compared with their synthetic precursors **3**. These suggested that compounds **5 **and **3 **probably have similar mechanism of action towards HIV-1 RT, and the NH unit in compounds **3** was not crucial to potent anti-HIV-1 RT activity.

The basic SAR prompted us to synthesize a series of *N-*alkyl analogues **5d** –**5t** with *ortho*-substituents R^1^ (Table 1). Compound **5i** (R^1^ = 2-Br, R^2^ = 3,5-difluorobenzyl) exhibited the highest enzyme activity. with a 88.89% inhibitory ratio against HIV-1 RT at the tested concentration, which increased nearly one fold from the precursor compound **3** (R^1^ = 2-Br, R^2^ = H). Compounds **5** were then tested for their syncytium reduction activities (EC_50_) and cytotoxities (CC_50_) in cell-based assays, in addition the selectivity index (SI) was calculated (Table 1). The most active compound was (*E*)-*N*-(2-bromostyryl)-*N*-3’,5’-difluorobenzylacetamide (**5i**, EC_50_ = 4 μm, SI = 29). Compound **5f** (IC_50_ = 320 μm, EC_50_ = 8 μm) is 40 times more potent in cell culture than in enzyme assays. One possibility for this discrepancy was supposed to be that the structure of compound **5f** was different from other *N*-alkylated compounds. In compound **5f**, two acetyl groups were substituted on the nitrogen atom, and this change in structure might cause compound **5f** active to other viral targets. Further structure modification of this kind of compounds is currently underway in our lab.

## 3. Experimental

### 3.1. General

Unless otherwise stated, all reagents were commercial analytical or chemical pure grades and were not additionally purified. IR spectra were measured on a Bio-Rad FTS-135 spectrometer (Bio-Rad, Richmond, CA) with KBr pellets, ν in cm^-1^; EI-MS data were recorded on an AutoSped 300 instrument; ^1^H-NMR spectra were recorded in CDCl_3_ on Bruker AM 400 or DRX-500 spectrometers. Chemical shifts are given in *δ* (ppm) with TMS as internal reference; column chromatography (CC): silica gel (200–300 mesh; Qingdao Marine Chemical In*c*.; Qingdao; China).

### 3.2. Preparation of (E)-β-phenylnitroolefins ***2***

An *ortho*-substituted benzaldehyde (2 mmol), ammonium acetate (0.1 g, 2.6 mmol) and nitromethane (3 mL) were placed in a 10 mL round bottomed flask equipped with a condenser. The mixture was refluxed and stirred for 2 hours and then evaporated to give a residue that was dissolved in CH_2_Cl_2_ (5 mL), washed successively with saturated brine and water, and the organic layer was evaporated to give the target compounds that were used without further purification (yields: 90–95%).

### 3.3. Preparation of (E)-N-phenylstyrylacetamides ***3***

Under a N_2_ atmosphere, the appropriate compound **2 **(1 mmol), iron powder (1.12 g, 20 mmol), acetic acid (0.2 mL) and acetic anhydride (6 mL) were added to a 20 mL round flask with a condenser. The mixture was refluxed for 2 hours and then cooled to room temperature. Methanol (20 mL) was added to dilute the mixture, which was filtered through Celite^®^. The filtrate was poured onto ice cooled water (50 mL) and then extracted with ethyl acetate. The organic layer was evaporated to a residue and dissolved in methanol (20 mL). The methanolic solution was adjusted to pH 12–14 with methanolic potassium hydroxide to hydrolyze diacetyl compounds **4 **to target compounds **3** and this basic solution was poured onto water (50 mL) and extracted again with ethyl acetate. The organic layer was washed successively with saturated brine and water, dried over anhydrous Na_2_SO_4_, and then evaporated to give a crude product which was purified on a silica gel column eluted with hexane/ethyl acetate (4:1, V/V) to give compounds **3** (yields: 45–50%). 

### 3.4. General procedure for the preparation of (E)-N-phenylstyryl-N-alkylacetamides ***5:*** Preparation of (E)-N-(3-bromostyryl)-N-methylacetamide *(**5a**)*

A solution of compound **3** (R^1^ = 3-Br, 98 mg, 0.5 mmol) in dry tetrahydrofuran (5 mL) was cooled to 0 °C, sodium hydride (0.6 mmol) was added, the mixture was kept stirring at 0°C for 30 min, then a solution of iodomethane (0.6 mmol) in tetrahydrofuran (1 mL) was added. The mixture was stirred at 0°C for another 30 min, then warmed up to ambient temperature and stirred until the completion of the reaction as monitored by TLC. The mixture was poured onto ice cooled water and extracted with ethyl acetate, the organic layer was then washed with brine and dried with anhydrous Na_2_SO_4_. After evaporation, the residue was purified on silica gel chromatography giving the target compound **5a** as a white powder; yield: 53%; ^1^H-NMR (CDCl_3_) *δ*: 8.11 (d, *J* = 14.9 Hz, 1H), 7.53–7.15 (m, 4H), 5.90 (d, *J* = 14.9 Hz, 1H), 3.25 (s, 3H), 2.36 (s, 3H); IR ν_max_ (KBr) cm^-1^: 1670, 1634; EI-MS *m/z* (%): 255 [M+2]^+^ (75), 253 [M]^+^ (79), 213 (95), 211 (100), 185 (13), 183(13), 131 (15). Compounds **5b–5t **(structures provided in Table 1) were synthesized using the above method.

*(E)-N-(4-bromostyryl)-N-methylacetamide* (**5b**): White powder, yield: 69%; ^1^H-NMR *δ*: 8.13 (d, *J* = 14.9 Hz, 1H), 7.43 (d, *J* = 8.2 Hz, 2H), 7.20 (d, *J* = 8.2 Hz, 2H), 5.92 (d, *J* = 14.9 Hz, 1H), 3.26 (s, 3H), 2.36 (s, 3H); IR ν_max_ cm^-1^: 1674, 1638; EI-MS *m/z* (%): 255 [M+2]^+^ (40), 253 [M]^+^ (43), 213 (90), 211 (100), 185 (10), 183(10), 168 (33), 149 (37), 131 (30), 117 (20).

*(E)-N-(2-bromostyryl)-N-methylacetamide* (**5c**): White powder, yield 50%; ^1^H-NMR *δ*: 8.00 (d, *J* = 15.0 Hz, 1H,), 7.55–7.00 (m, 4H), 6.22 (d, *J* = 15.0 Hz, 1H), 3.24 (s, 3H), 2.28 (s, 3H); IR ν_max_ cm^-^^1^: 1680, 1632; EI-MS *m/z* (%): 255 [M+2]^+^ (25), 253 [M]^+^, 213 (50), 211 (50), 185 (55), 183(55), 132 (100), 117 (67).

*(E)-N-(2-bromostyryl)-N-ethylacetamide* (**5d**): White powder, yield 65%; ^1^H-NMR *δ* : 7.85 (d, *J* = 14.6 Hz, 1H), 7.54–7.02 (m, 4H), 6.27 (d, *J* = 14.6 Hz, 1H), 3.82 (q, *J* = 7.0 Hz, 2H), 2.28 (s, 3H), 1.31 (t, *J* = 7.0 Hz, 3H); IR ν_max_ cm^-1^: 1677, 1632, 1399, 1246; EI-MS *m/z* (%): 268 [M+2]^+^ (45), 267 [M] (45), 227 (75), 225 (76), 210 (38), 188 (32), 130 (100).

*(E)-N-(2-bromostyryl)-N-allylacetamide* (**5e**): Viscous oil, yield 65%; ^1^H-NMR *δ*: 7.95 (d, *J* = 14.2 Hz, 1H), 7.55-7.04 (m, 4H), 6.33 (d, *J* = 14.2 Hz, 1H), 5.86-5.83 (m, 1H), 5.31-5.02 (m, 2H), 4.43-4.29 (m, 2H), 2.24 (s, 3H); IR ν_max_ cm^-1^: 1678, 1632, 1395, 1225; EI-MS *m/z* (%): 281 [M+2]^+^ (90), 279 [M] (100), 239 (87), 237 (94), 224 (40), 222 (48), 158 (80), 156 (37), 143 (27), 130 (51), 117 (83).

*(E)-N-(2-bromostyryl)-N-acetylacetamide* (**5f**): White powder, yield 55%; ^1^H-NMR *δ*: 7.56 (d, *J* = 7.9 Hz, 2H), 7.30 (t, *J* = 7.4 Hz, 1H), 7.16 (t, J = 7.7 Hz, ), 6.85 (d, *J* = 14.4 Hz, 1H), 6.76 (d, *J* = 14.4 Hz, 1H), 2.44 (s, 6H); IR ν_max_ cm^-1^: 1715, 1644, 1468, 1426, 1368, 1274, 1228; FAB-MS *m/z* (%): 282 [M+1]^+^ (100).

*(E)-N-(2-bromostyryl)-N-benzylacetamide* (**5g**): White powder, yield 67%; mp 86-88 °C; ^1^H-NMR *δ*: 8.08 (d, *J* = 14.3 Hz, 1H), 7.48-7.02 (m, 9H), 6.30-6.19 (m, 1H), 5.03, (s, 2H), 2.43 (s, 3H); IR ν_max_ cm^-1^: IR ν_max_ cm^-1^: 1663, 1578, 1398, 1322, 1256, 1229; EI-MS *m/z* (%): 331 [M+2]^+^ (20), 329 [M] (22), 289 (23), 287 (25), 250 (8), 208 (10), 91 (100).

*(E)-N-(2-bromostyryl)-N-2’,6’-difluorobenzylacetamide* (**5h**): White powder, yield 70%; ^1^H-NMR *δ*: 7.50-6.80 (m, 8H), 6.27 (d, *J* = 14.2 Hz, 1H), 5.20 (s, 2H), 2.38 (s, 3H); IR ν_max_ cm^-1^: 1669, 1627, 1469, 1406, 1316. EI-MS *m/z* (%): 367 [M+2]^+^ (15), 365 [M]^+^ (15), 325 (35), 323 (27), 286 (7), 244 (15), 127 (100).

*(E)-N-(2-bromostyryl)-N-3’,5’-difluorobenzylacetamide* (**5i**): White powder, yield 66%; ^1^H-NMR *δ*: 7.52-6.67 (m, 8H), 6.17 (d, *J* = 14.5 Hz, 1H), 5.00 (s, 2H), 2.43 (s, 3H); IR ν_max_ cm^-1^: 160, 1635, 1598, 1461, 1437, 1396, 1319; EI-MS *m/z* (%): 367 [M+2]^+^ (45), 365 [M]^+^ (48), 325 (83), 323 (90), 286 (20), 244 (57), 127 (100). 117 (95).

*(E)-N-(2-bromostyryl)-N-2’,6’-dichlorobenzylacetamide* (**5j**): White powder, yield 67%; ^1^H-NMR *δ*: 7.53-6.94 (m, 8H), 6.21 (d, J = 12.9 Hz, 1H), 5.35 (s, 2H), 2.35 (s, 3H); IR ν_max_ cm^-1^: 1674, 128, 1580, 1435, 1402, 1340; EI-MS *m/z* (%): 401 [M+4]^+^ (10), 399 [M+2]^+^ (20), 397 [M]^+^ (15), 357 (40), 322 (64), 320 (58), 161 (68), 159 (100) 117 (42).

*(E)-N-(2-chlorostyryl)-N-methylacetamide* (**5k**): White powder yield: 67%; ^1^H NMR *δ*: 8.07 (d, *J* = 14.9 Hz, 1H), 7.43-7.13 (m, 4H), 6.27 (d, *J* = 14.9 Hz), 3.26 (s, 3H); 2.30 (s, 3H); IR ν_max_ cm^-1^: 1680, 1632, 1387, 1332, 1256; EI-MS *m/z* (%): 211 [M+2]^+^ (25), 209[M]^+^ (75), 169 (32), 167 (100), 132 (100), 130(35).

*(E)-N-(2-chlorostyryl)-N-ethylacetamide* (**5l**): White powder, yield: 73%; ^1^H-NMR *δ*: 7.89 (d, *J* = 14.6 Hz, 1H), 7.39-7.07 (m, 4H), 6.29 (d, *J* = 14.6 Hz, 1H), 3.81(q, *J* = 6.9 Hz, 2H), 2.27 (s, 2H), 1.29 (t, J = 6.9 Hz, 3H); IR ν_max_ cm^-1^: 1677, 1633, 1440, 1399, 1246; EI-MS *m/z* (%): 225 [M+2]^+^ (28), 223 [M]^+^ (85), 183 (33), 181 (100), 168 (24), 166 (68), 146 (32), 130 (64).

*(E)-N-(2-chlorostyryl)-N-allylacetamide* (**5m**): White powder, yield: 63%; ^1^H-NMR *δ*: 8.00 (d, *J* = 14.5 Hz, 1H), 7.51-7.07 (m, 4H), 6.34-6.26 (m, 1H), 5.91-5.60 (m, 1H), 5.28-5.19 (m, 2H), 4.26 (d, *J* = 5.0 Hz, 2H), 2.32 (s, 3H); IR ν_max_ cm^-1^: 1674, 1632, 1440, 1397; EI-MS *m/z* (%): 237 [M+2]^+^ (5), 235 [M]^+^ (14), 193 (10), 178 (12), 141 (100), 139 ( 87), 77 (100).

*(E)-N-(2-fluorostyryl)-N-methylacetamide* (**5n**): White powder; yield: 70%; ^1^H-NMR *δ*: 8.12 (d, *J* = 15.0 Hz, 1H), 7.51–7.03 (m, 4H), 6.07 (d, *J* = 15.0 Hz, 1H), 3.23 (s, 3H); 2.30 (s, 3H); IR ν_max_ cm^-^^1^: 1671, 1637, 1488, 1457, 1385, 1314; EI-MS *m/z* (%): 193 [M]^+^ (60), 151 (100), 109(50). 

*(E)-N-(2-fluorostyryl)-N-allylacetamide* (**5o**): White powder, yield: 63%; ^1^H-NMR *δ*: 8.10 (d, *J* = 14.7 Hz, 1H), 7.44-7.01 (m, 4H), 6.07-6.00 (m, 1H), 5.90-5.81 (m, 1H), 5.28-5.04 (m, 2H), 4.27 (d, *J* = 5.0 Hz, 2H), 2.34 (s, 3H); IR ν_max_ cm^-1^: 1679, 1636, 1490, 1395; EI-MS *m/z* (%): 219 [M]^+^ (78), 177 (67), 162 (57), 148 ( 28), 135 (35), 109 (100), 101 (26), 68 (72).

*(E)-N-(2-methylstyryl)-N-methylacetamide* (**5p**): White powder, yield: 65%; ^1^H-NMR *δ*: 7.97 (d, *J* = 15.0 Hz, 1H), 7.45–7.12 (m, 4H), 6.09 (d, *J* = 15.0 Hz), 3.24 (s, 3H); 2.35 (s, 3H); 2.29 (s, 3H); IR ν_max_ cm^-1^: 1668, 1633, 1468, 1440, 1382, 1310, ; EI-MS *m/z* (%): 189 [M]^+^ (67), 147 (100), 132 (20), 106 (30). 73 (57).

*(E)-N-(2-methylstyryl)-N-allylacetamide* (**5q**): White powder, yield: 61%; ^1^H-NMR *δ*: 7.90 (d, *J* = 14.7 Hz, 1H), 7.45-7.07 (m, 4H), 6.18-6.11 (m, 1H), 5.87-5.84 (m, 1H), 5.28-5.18 (m, 2H), 4.44 (d, *J* = 5.0 Hz, 2H), 2.33 (s, 3H), 2.30 (s, 3H); IR ν_max_ cm^-1^: 1675, 1635, 1396; EI-MS *m/z* (%): 215 [M]^+^ (100), 173 (42), 158 (53), 130 (44), 117 (46), 105 (41) 68 (42).

*(E)-N-(2,4-dimethoxystyryl)-N-methylacetamide* (**5r**): White powder, yield: 73%; ^1^H-NMR *δ*: 7.90 (d, *J* = 15.0 Hz, 1H), 7.39-7.18 (m, 2H), 6.47 (s, 1H), 6.11 (d, *J* = 15.0 Hz, 1H), 3.85, 3.81 (s, each 3H), 3.21 (s, 3H), 2.28 (s, 3H); IR ν_max_ cm^-1^: 1672, 1634, 1580, 1508, 1461, 1416, 1390, 1332, 1288; EI-MS *m/z* (%): 235 [M]^+^ (100), 193 (20), 192 (20), 178 (30), 161 (30), 137 (22).

*(E)-N-(2,4-dimethoxystyryl)-N-ethylacetamide* (**5s**): White powder, yield: 69%; ^1^H-NMR *δ*: 7.85 (d, *J* = 14.7 Hz, 1H), 7.23-7.17 (m, 2H), 6.46 (s, 1H), 6.15 (d, *J* = 14.7 Hz, 1H), 3.83, 3.80 (s, each 3H), 3.68 (q, *J* = 6.7 Hz, 2H), 2.24 (s, 3H), 1.19 (t, J = 6.7 Hz, 3H); IR ν_max_ cm^-1^: 1668, 1633, 1608, 1579, 1505, 1462, 1402; EI-MS *m/z* (%): 249 [M]^+^ (100), 207 (15), 206 (19), 175 (17), 160 (13), 151 (15), 70 (57).

*(E)-N-(2,4-dimethoxystyryl)-N-allylacetamide* (**5t**): White powder, yield: 65%; ^1^H-NMR *δ*: 7.94 (d, *J* = 15.1 Hz, 1H), 7.34-7.15 (m, 2H), 6.45 (s, 1H), 6.15 (d, *J* = 15.1 Hz, 1H), 5.87-5.80 (m, 1H), 5.25-5.10 (m, 2H), 3.39 (d, *J* = 4Hz, 2H), 3.83, 3.73 (s, each 3H), 2.29 (s, 3H); IR ν_max_ cm^-1^: 1671, 1634, 160, 1579, 1505, 1398; EI-MS *m/z* (%): 261 [M]^+^ (92), 221 (45), 219 (53), 177 (42), 162 (41), 147 (81), 109 (42), 73 (100).

### 3.5. Biological activity assays

Compounds **5** were assayed for their anti-HIV-1 activities as reported previously [14,15,16].

*HIV-1 reverse transcriptase assay*: The inhibition of compounds **5** on recombinant HIV-1 RT activity was determined with a commercially available ELISA kit (Roche Molecular Biochemicals). The compounds were incubated with DIG-labeled-reaction mixture at 37 °C for 15 h, and then anti-DIG-POD solution was added, followed by substrate ABTS. Foscarnet was used as a positive compound. The absorbance at 405 nm/490 nm (A405/490) was read in the ELISA reader mentioned above. Inhibition ratio on recombinant RT activity was determined from the dose–response curve.

*Cell based anti-HIV-1 bioassay*: C8166 cells were maintained in RPMI-1640 supplemented with 10% heat inactivated newborn calf serum (Gibco), The cells used in all experiments were in log-phase growth. 3′-Azido-3′-deoxythymidine (AZT), the positive control, was purchased from Sigma (USA).

*Syncytium reduction assay*: In the presence of 100 µL of various concentrations of compounds, C8166 cells (4 × 10^5^ mL^-1^) were infected with HIV-1_IIIB _at a multiplicity of infection (MOI) of 0.06. The final volume per well was 200 µL. AZT was used as a positive control. After 3 days of culture, the cytopathic effect (CPE) was measured by counting the number of syncytia (multinucleated giant cell) in each well under an inverted microscope. Percentage inhibition of syncytial cell number in treated culture to that in infected control culture and 50% effective concentration (EC_50_) was calculated.

*Cytotoxicity assay*: The cellular toxicity of compounds on C8166 cells was assessed by MTT methods. Briefly, cells were seeded on a microplate in the absence or presence of various concentrations of compounds in triplicate and incubated at 37 °C in a humid atmosphere of 5% CO_2 _for 72 h. The supernatants were discarded and MTT reagent (5 mg/mL in PBS) was added to each wells, then incubated for 4 h, 100 µL of 50% DMF-20% SDS was added. After the formazan was dissolved completely, the plates were read on a Bio-Tek ELx 800 ELISA reader at 595/630 nm. The cytotoxic concentration that caused the reduction of viable cells by 50% (CC_50_) was calculated from dose-response curve.

## 4. Conclusions

We have synthesized a series of (*E*)-*N-*phenylstyryl*-N-*alkylacetamides. Evaluation of the title compounds as NNRTIs of HIV-1 *in vitro* showed that compound **5c** showed a higher TI value than its isomer **5a**, which suggested that higher enzymatic inhibitory ratio would lead to increased anti-HIV activity in cell-based assay. Compound **5i** exhibited the highest inhibition activity in both enzyme and cell based assays. Although the TI values of the tested compounds were suboptimal, the title compounds possessed low molecular weight and contained a naturally occurring structure unit (hamigeroxalamic acid). The SAR exploration provided us a guide for further anti-HIV-1 study of the derivatives in this family. Compound **5i** represented a new lead for the design and synthesis of more potent and selective analogues act as NNRTIs. Further structure modification and anti-HIV-1 activities of compounds **5 **are underway.
